# Dual mTORC1/C2 inhibitors suppress cellular geroconversion (a senescence program)

**DOI:** 10.18632/oncotarget.4836

**Published:** 2015-07-27

**Authors:** Olga V. Leontieva, Zoya N. Demidenko, Mikhail V. Blagosklonny

**Affiliations:** ^1^ Department of Cell Stress Biology, Roswell Park Cancer Institute, Buffalo, NY, USA

**Keywords:** Gerotarget, aging, senescence, rapamycin, rapalogs, pan-mTOR inhibitors, Pathology Section

## Abstract

In proliferating cells, mTOR is active and promotes cell growth. When the cell cycle is arrested, then mTOR converts reversible arrest to senescence (geroconversion). Rapamycin and other rapalogs suppress geroconversion, maintaining quiescence instead. Here we showed that ATP-competitive kinase inhibitors (Torin1 and PP242), which inhibit both mTORC1 and TORC2, also suppressed geroconversion. Despite inhibition of proliferation (in proliferating cells), mTOR inhibitors preserved re-proliferative potential (RP) in arrested cells. In p21-arrested cells, Torin 1 and PP242 detectably suppressed geroconversion at concentrations as low as 1-3 nM and 10-30 nM, reaching maximal gerosuppression at 30 nM and 300 nM, respectively. Near-maximal gerosuppression coincided with inhibition of p-S6K(T389) and p-S6(S235/236). Dual mTOR inhibitors prevented senescent morphology and hypertrophy. Our study warrants investigation into whether low doses of dual mTOR inhibitors will prolong animal life span and delay age-related diseases. A new class of potential anti-aging drugs can be envisioned.

## INTRODUCTION

Aging is driven by conservative genetic pathways [1–21]. These pathways converge at TOR (target of rapamycin) [22–26]. Allosteric mTOR inhibitors known as rapalogs such as rapamycin (Sirolimus), everolimus, deforolimus and temsirolimus are available in the clinic [27–33].

Rapamycin and other rapalogs inhibit some, but not all, mTOR functions [34–43]. Rapalogs bind FKBP12 and this complex in turn inhibits mTOR complex 1 (mTORC1). In most conditions, rapamycin does not inhibit mTOR complex 2 (mTORC2). Inhibition of phosphorylation of mTORC1 substrates by rapamycin is concentration-dependent [41]. At clinically-relevant concentrations, rapamycin does not inhibit phosphorylation of 4EBP1 at T37/46, while inhibiting S6K/S6 pathway [34–44]. In contrast, direct inhibitors of the TOR kinase (non-rapalogs) abrogate all activities of mTORC1 and mTORC2 [39, 45–48]. These ATP-competitive inhibitors of the TOR kinase are not selective and at higher concentrations inhibit PI3K, ATM and some other kinases.

It is difficult to predict the outcome of life-long administration of dual mTOR inhibitors in animals. On one hand, they inhibit both rapamycin-sensitive and -insensitive functions of mTOR and thus might extend life span extensively. Yet, the role of rapamycin-insensitive functions in aging is unknown. Furthermore, potential toxicity due to lack of selectivity toward TOR may preclude antiaging effects. Fortunately, a simple cellular model is available to investigate anti-aging activity in cell culture. In proliferating cells, mTOR is active and drives cellular growth. When the cell cycle is forcefully arrested, but mTOR is still active (as in proliferating cells), then mTOR drives “senescence” [49, 50].

In other words, mTOR drives conversion from reversible cell cycle arrest to senescence (geroconversion) and rapamycin suppresses or decelerates geroconversion [51–79].

Senescent cells cannot restart proliferation (re-proliferation), even when the arrest is lifted and cells re-enter the cell cycle. In the presence of active mTOR, p21- or p16-arrested cells lose their re-proliferative potential (RP). In the presence of rapamycin [54, 55, 62] and everolimus [61], cells maintain RP during cell cycle arrest and can re-proliferate, when the arrest is lifted. Also, rapalogs moderately suppress cellular hypertrophy and beta-Gal-positive senescent morphology. Here we tested whether non-rapalog mTOR inhibitors such as Torin 1 and PP242 can suppress geroconversion.

## RESULTS

### mTOR inhibitors suppress geroconversion

**Figure 1:**
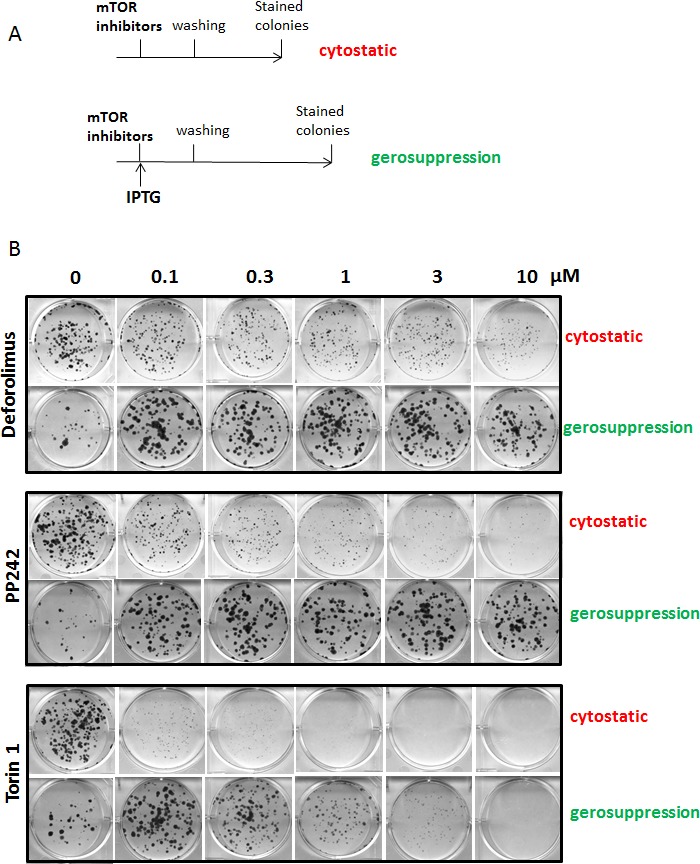
Cytostatic and gerosuppressive effects **A**. Schema of experiment; **B**. Cytostatic and gerosupressive effects of deforolimus, Torin 1 and PP242 in HT-p21 cells. Cells were treated with serial dilutions of deforolimus, PP242 or Torin 1 as depicted in schema in (A). To determine cytostatic activity, cells were treated with drugs for 3 days, then drugs were washed out and cells were allowed to recover in drug-free medium. Colonies were stained after 5 days of regrowth. To investigate drug effects on geroconversion, HT-p21 cells were treated with IPTG in the presence of serial dilutions of the drugs. After 3-day treatment, drugs were washed out and colonies were allowed to form for 9 days, then stained and counted in triplicates. Data are mean ± SD.

We compared gerosuppressive and anti-proliferative (cytostatic) effects of dual mTOR inhibitors, Torin 1 and PP242 (Fig. 1A). We also tested deforolimus, a rapalog also known as ridaforolimus. To measure cytostatic effects, proliferating HT-p21 cells were treated with these inhibitors (Fig. 1). After a 3-day treatment, drugs were washed out and cells were incubated in fresh medium for another 3-6 days. Colonies were stained with Crystal Violet. TOR inhibitors decreased size of colonies but not their number. This is consistent with reversible inhibition of proliferation in the presence of inhibitors (Fig. 1). Cells retained their re-proliferative potential and resumed proliferation when the inhibitors were removed. Therefore, the number of colonies remained the same as in control but their size became progressively smaller with increasing concentrations of inhibitors (Fig.1, see “cytostatic” panels).

To investigate effects of the drugs on geroconversion, we induced ectopic p21 in HT-p21 cells by treating them with IPTG [62, 80, 81]. These cells undergo senescence when p21 is turned on by IPTG and lose their re-proliferative potential (RP). When IPTG is washed out, the senescent cells can re-enter the cell cycle but cannot proliferate [63].

Suppression of geroconversion was evaluated by the ability of IPTG-treated HT-p21 cells to resume proliferation after IPTG was removed (Fig. 1, gerosuppression). Treatment with IPTG alone decreased the number of colonies, (Fig. 1B; no drug). This is consistent with irreversible senescence in most IPTG-treated cells, so only a few cells were quiescent and retained RP. Treatment of IPTG-arrested cells with deforolimus, Torin 1 or PP242 increased the number of colonies. These colony-forming cells were in quiescent state and retained potential to re-proliferate. Cells resumed proliferation once IPTG and drugs were removed. In summary, the effects of mTOR inhibitors on proliferation (cytostatic effect) and re-proliferative potential (gerosuppressive effect) were opposite. While decreasing the size of colonies in first case, mTOR inhibitors increased the number of colonies in second case (Fig. 1B).

**Figure 2:**
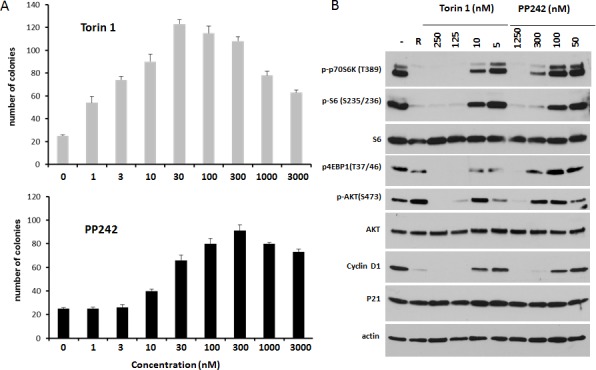
Gerosuppression and mTOR inhibition **A**. Geroconversion. Torin 1 and PP242 were added to IPTG-treated HT-p21 cells, as shown in Figure 1A. Cells plated at low density were treated with IPTG in the presence of serial dilutions of inhibitors. After 3 days, IPTG and inhibitors were washed out and cells were incubated in drug-free medium. Then colonies were stained with Crystal Violet and counted. Data are mean number of colonies per a sector of a well ± SD. **B**. Immunoblot analysis. HT-p21 cells were treated with IPTG in combination with Torin 1 or PP242 for 24 h and lysed. Rapamycin (R) at 500 nM was included as additional control. Immunoblotting was performed with the indicated antibodies. Note: IPTG-induced p21 is highly expressed in all samples, confirming that the inhibitors do not interfere with p21 induction by IPTG and cell cycle arrest.

At concentrations shown in Fig. 1B, gerosuppressive effect of Torin 1 was maximal at the lowest concentration shown (100 nM). At higher concentrations (> 300 nM), the gerosuppressive effect of Torin 1 was partially lower (Torin 1 partially canceled its gerosuppressive effect) and colonies became smaller. Therefore, we next titrated concentrations down (Fig. 2A). IPTG-arrested HT-p21 cells were treated with a range of concentrations from 1 nM to 3000 nM, according to experimental schema shown in Fig. 1A. Gerosuppressive effect of Torin 1 was shifted toward lower concentrations compared to that of PP242 (Fig. 2A). Minimal concentrations that suppressed geroconversion were 1 nM and 10 nM for Torin 1 and PP242, respectively. Maximum effects of Torin 1 and PP242 were observed at concentrations 30 nM and 300 nM, respectively, indicating that Torin 1 is overall 10 times more potent than PP242. Therefore, 10-fold lower concentrations of Torin 1 than PP242 could be used to achieve equipotent effect.

### Correlation between gerosuppression and mTOR inhibition

We next investigated inhibition of the mTOR pathway by Torin 1 and PP242. Consistent with gerosuppressive effects (Fig. 2A), Torin 1 inhibited phosphorylation of S6 kinase (target of mTORC1) and its downstream target phospho-S6 at concentrations 10 times lower than PP242 (Fig. 2B). The inhibition of the S6K/S6 axis corresponded to concentrations at which preservation of RP (gerosuppression) was observed. The S6K/S6 axis is a major target of rapamycin.

At higher concentrations, at which gerosuppression was already near-maximal, Torin 1 and PP242 exerted rapamycin-resistant effects: namely, inhibition of phosphorylation of 4EBP1 (T37/46) and Akt (S473). Equipotent concentrations were approximately 10 times higher for PP242 than for Torin 1.

Finally, like rapamycin, Torin 1 and PP242 did not decrease p21 levels, so they did not abrogate IPTG-induced arrest. At equipotent concentrations both mTOR inhibitors decreased cyclin D1, a marker of senescent hyper-mitogenic cell cycle arrest [63]. Hyper-elevated cyclin D1 is a common marker of senescent phenotype [63].

### mTOR kinase inhibitors prevent senescent morphology

**Figure 3:**
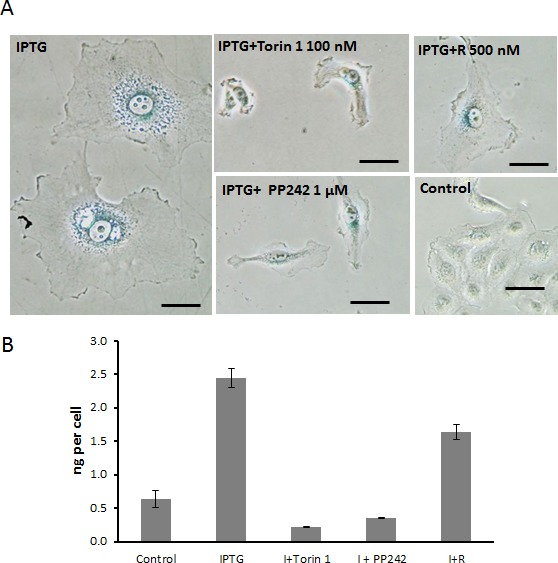
Torin 1 and PP242 prevent senescent morphology and hypertrophy in HT-p21 cells induced to senesce by IPTG **A**. Cells were treated with IPTG (I) in the presence of Torin 1, PP242 or Rapamycin (R) for 4 days and stained for beta-Gal. Bar – 100 μm. **B**. Cells were treated with IPTG in combination with Torin 1 (100 nM), PP242 (1000 nM) or rapamycin (500 nM). After 3 days, cells were counted and lysed. Protein concentrations were measured. Data present mean ± SD of ng of protein per cell.

Treatment with IPTG resulted in a large flat cell morphology and beta-Gal-positivity (Fig. 3A). Enlarged cell size was due to hypertrophy as indicated by increased amount of protein per cell (Fig. 3B, compare control and IPTG). Torin 1 or PP242 prevented senescent morphology and decreased cell size. As it has been previously shown, rapamycin partially decreased beta-Gal staining. Rapamycin only partially prevented senescent morphology and hypertrophy (Fig. 3). Notably, Torin 1 and PP242 were far more potent in suppressing senescent morphology and hypertrophy, compared with rapamycin.

### Dual inhibitors of TORC1/2 prevent etoposide- and doxorubicin-induced senescence in normal human fibroblasts WI38t

**Figure 4:**
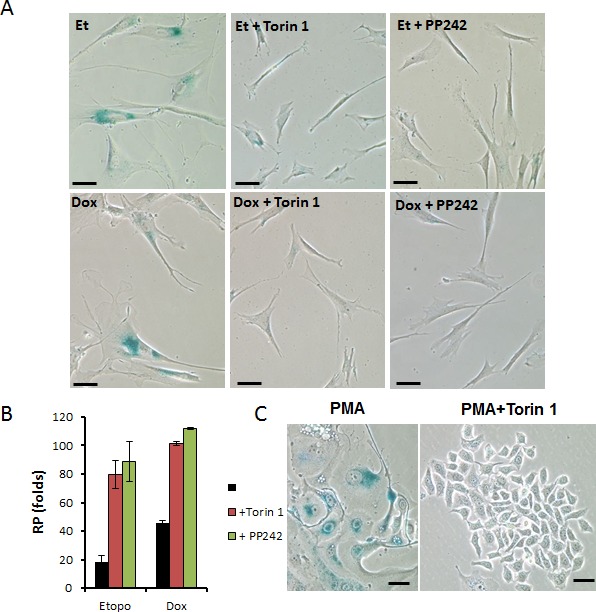
Torin 1 and PP242 suppress geroconversion in human WI38t fibroblasts and SkBr3 cells **A**. WI38t cells were treated with either 1 μg/ml etoposide or 50 ng/ml doxorubicin in the absence or presence of 100 nM Torin 1 or 1 μM PP242 for 4 days and stained for beta-Gal. Bar – 100 μm. **B**. Cells were treated as in (A). After 4-day treatment, cells were extensively washed to remove the drugs and allowed to regrow in drug-free medium and counted after 3 weeks in culture. RP (re-proliferative potential) was calculated by dividing final cell numbers by initially-plated number of cells. Data present mean ±SD from triplicate wells. C. Effect of Torin 1 on senescent morphology of SKBR3 cells undergoing senescence induced by PMA. Cells were pre-treated with Torin 1 (100 nM) for 24 h before addition of 100 nM PMA. After 3-day treatment with PMA, drugs were washed out, cells were incubated in drug-free medium for another 3 days and stained for beta-gal. Bar – 100 μm.

To investigate whether gerosuppressive activity of Torin 1 and PP242 is not cell-type specific, we next examined their effect on senescent morphology and RP in WI38t fibroblasts undergoing senescence by treatment with low concentrations of etoposide or doxorubicin. As shown in Fig. 4A, both etoposide and doxorubicin treatment caused large flat and beta-gal positive morphology in WI38t cells. In etoposide- and doxorubicin-treated cells, co-treatment with either Torin 1 or PP242 prevented senescent morphology. Furthermore, when WI38t cells were pre-treated with either senescing-drug (etoposide or doxorubicin) alone, most of the cells lost the ability to re-proliferate after drugs were removed. Co-treatment of cells with senescing-drugs and either Torin 1 or PP242 preserved RP after removal of the drugs. In other words, treatment of cells with etoposide in the presence of either Torin 1 or PP242 preserved some cells in reversible quiescence instead of irreversible senescence. mTOR inhibitors also prevented senescent morphology of SKBR3 cells undergoing senescence by treatment with PMA (Fig. 4C), a model of senescence described previously [82].

## DISCUSSION

Rapamycin and other rapalogs are anti-inflammatory and anti-aging drugs [83, 84]. Rapamycin slows down aging, prevents age-related diseases and extends life span in all species tested, including mice [85–104].

On cellular level, rapamycin and other rapalogs inhibit geroconversion from cell cycle arrest to senescence [51, 50]. In the organism, geroconversion drives hyperfunctions, slowly causing age-related pathologies [22, 105–107]. Suppression of geroconversion (gerosuppression) by rapalogs is a surrogate of their anti-aging effects. Therefore, to evaluate whether dual mTOR inhibitors are potential anti-aging drugs, we investigated gerosuppression in cell culture.

Loss of re-proliferative potential (RP) is a quantitative marker of geroconversion. Non-senescent (quiescent) cells retain RP, meaning that they can re-start proliferation (re-proliferation), when arrest is reversed. In contrast, senescent cells lack RP. We found that in the presence of Torin 1 and PP242, arrested cells retained RP. Like rapalogs, these pan-mTOR inhibitors prevented loss of RP during cell cycle arrest caused by p21 and etoposide. Importantly, mTOR inhibitors suppressed geroconversion despite their intrinsic anti-proliferative effect (in proliferating cells).

The ability of mTOR inhibitors to preserve RP indicates that (at gerosuppressive concentrations) mTOR inhibitors are not harmful in any way; otherwise cells would not resume proliferation and would not form colonies. In fact, at high concentrations, the gerosuppressive effect, measured as preservation of RP, decreased and even disappeared.

Gerosuppression coincided with inhibition of the mTORC1/S6K/S6 axis. The maximal gerosuppression, as measured by RP, was similar for rapalogs and pan-mTOR inhibitors.

PP242 was 10 times less potent but it was also less cytostatic and inhibited mTORC1/S6K/S6 at 10-fold higher concentrations. We conclude that their effects are almost identical at equipotent concentrations. At high concentrations, Torin 1 and PP242 were more efficient than rapamycin in suppressing cellular hypertrophy and senescent morphology. This indicates that morphology and hypertrophy depend in part on rapamycin-insensitive functions of mTOR.

Rapamycin and other rapalogs have been safely used even in daily high doses for many years in patients [108–110], so their use as anti-aging drugs (intermittent schedules) is expected to be without significant side effects. Dual mTOR inhibitors have all activities of rapamycin plus other effects and therefore must have more side effects by definition. Still, our study indicates that, at low concentrations, they may be considered as potential anti-aging drugs because they can preserve re-proliferation, which would be impossible if the drugs were toxic, given the delicate mechanism of cellular division.

Still, mTOR inhibitors are cytostatic. Therefore, for their clinical development as anti-aging drugs, it would be important to determine balance between cytostatic vs gerosuppressive effects. mTOR inhibitors also vary in their affinity to mTORC1 and mTORC2 complexes, as well as by their off-target effects. Therefore, a larger variety of inhibitors should be further tested at a wider range of concentrations for their effects on cell proliferation vs geroconversion. Optimal gerosuppressive concentrations of various mTOR inhibitors should be selected (Oncoscience, 2015, in press).

## MATERIALS AND METHODS

### Cell lines and reagents

HT-p21 cells, derived from HT1080 human fibrosarcoma cells, in which p21 expression can be turned on and off using IPTG (isopropyl-thio-galactosidase) were described [80, 81, 62]. HT-p21 cells were cultured in DMEM/10% FC2 serum (HyClone FetaClone II; HyClone Laboratories, Inc, Logan, UT), Immortalized WI38t human fibroblasts, described previously [111], and SKBR3, breast adenocarcinoma cell line (ATCC, Manassas, VA), were maintained in DMEM/10% FBS. Rapamycin was purchase from LC Laboratories (Woburn, MA). Torin 1 and PP242 were from Selleckchem (Houston, TX). Stock solutions were prepared in DMSO.

### Re-proliferative potential (RP)

Cells were plated at low densities and treated with senescence-inducing agents as described in figure legends. After 3-4-day treatment drugs were washed out and cells were allowed to regrow in drug-free medium for a few days as indicated in figure legends. Colonies were stained with 1% Crystal Violet (Sigma-Aldrich) and counted.

### Immunoblot analysis

Whole cell lysates were prepared using boiling lysis buffer (1%SDS, 10 mM Tris.HCl, pH 7.4). Equal amounts of proteins were separated on 10% or gradient polyacrylamide gels and transferred onto PVDF membranes [111]. Primary antibodies for the following proteins were used: rabbit antibodies for phospho-p70S6K(T389), phospho-pS6(S235/236), phospho-4EBP1(T37/46), phospho-AKT(S473), AKT and mouse antibody for S6 – from Cell Signaling Technology (Danvers, MA); mouse antibodies for cyclin D1 and p21 were from Santa Cruz Biotechnology (Paso Robles, CA) and BD Biosciences (San Jose, CA), respectively. Actin antibody was purchased from Sigma-Aldrich (St. Louis, MO).

Secondary anti-mouse and anti-rabbit antibodies were from Cell Signaling.

### SA-β-Gal staining

β-gal staining was performed using Senescence-galactosidase staining kit (Cell Signaling Technology), according to manufacturer's protocol. Cells were microphotographed under light microscope.
